# Zearalenone, an abandoned mycoestrogen toxin, and its possible role in human infertility

**DOI:** 10.18502/ijrm.v20i2.10507

**Published:** 2022-03-21

**Authors:** Abbas Ali Jafari-Nodoushan

**Affiliations:** Department of Medical Parasitology and Mycology, School of Medicine, Shahid Sadoughi University of Medical Sciences, Yazd, Iran.

##  Dear Editor,

Mycotoxins, the secondary metabolites produced by several common airborne filamentous fungi, may contaminate human food and animal feeds, especially in developing countries (1, 2). Even though aflatoxin has been widely studied and reported as the most common and important mycotoxin in global and Iranian scientific literature (3, 4), zearalenone (ZEA) has sadly been abandoned, particularly in Iran.

**Figure 1:**
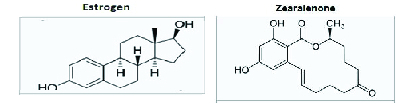
Zearalenone and estrogen molecular structures (7).

ZEA, which is also known as F-2 toxin, ecoestrogen and mycoestrogen, is an estrogenic-like fungal toxin (Figure 1) produced by several *Fusarium (F.) *species such as *F. graminearum, F. culmoru, F. cerealis, F. equiseti, F. verticillioides,* and *F. incarnatum* (5). These fungi commonly contaminate several cereal grains, such as corn (in particular), barley, oats, rye, wheat, rice, sorghum, and their derivatives worldwide (6).

This mycotoxin can also contaminate meat and dairy commodities, and can even enter into the aquatic environment via rain, which can affect marine and human health in various ways (8). ZEA is a heat stable toxin, which does not get broken down during usual food processing and causes common health problems (9). ZEA can interfere with DNA and protein synthesis, result in lipid peroxidation, and cause cytotoxicity by motivating germ cell apoptosis. ZEA (especially α-ZEA) competitively pairs with the estrogen receptor, resulting in reproductive system dysfunction via the estrogen signaling pathway (10, 11).

ZEA and its derivatives can affect mammalian reproductive capacity via the control of sex hormone synthesis; it acts similarly to estrogen molecules to interfere with the production of follicle-stimulating hormone and luteinizing hormone. This fungal toxin also can disrupt the expression and actions of the related steroidogenic enzymes (12).

In females, ZEA can cause hyperestrogenism, leading to major side effects. A study on prepubertal swine (known as the most sensitive species) showed common signs of hyperestrogenism including vulva inflammation, increased uterine size and secretions, mammary gland hyperplasia, increased rates of pseudo-pregnancy, and infertility. This toxin (with 1-5 PPM) also affects animals' reproductive systems, causing vulvovaginitis in females and feminization of male swine. It also interferes with conception, ovulation, implantation, and fetal development (13).

ZEA and its metabolites, known as xenoestrogens, tend to collect in human reproductive organs as a result of their high affinity for estrogen receptors, causing the proliferation of tumor cells and the development of cancer in the reproductive organs. Some reports have also shown links with several other disorders including deleterious effects on genital organs (because of increasing the proliferation of uteri corpus tissue), sexual serum hormones and oxidative stress following experimental feeding of post-weaning piglets with food containing 1.05 mg/kg of ZEA (14, 15).

No studies have as yet reported on the effect of ZEA on human ovulation. However, an in vivo study was conducted on the hormonal properties of ZEA in ruminant female animals which showed a reduction in their ovulation rates (16).

In humans, studies on ZEA show that it promotes germ cell apoptosis and permeability, which results in prematurity and other endocrine disorders in the female reproductive system and cycle. There are rare reports of possible male infertility due to ZEA intake via contaminated foods; for instance, ZEA can cause a reduction in testosterone, testicle weight and spermatogenesis (10). A study on the effect of ZEA on circulating testosterone concentration, testicular and epididymal morphology and sperm characteristics showed only a temporary and reversible effect on wild boar sperm motility (17). An investigation on healthy-weight and obese female mice which were exposed to ZEA (40 μg/kg) showed that this toxin can induce adverse ovarian function in obese mice (which spent less time at proestrus and more time in the metestrus/diestrus phase), suggesting that obesity can enhance ovarian sensitivity to ZEA exposure (18).

Many studies regarding food contamination with fungi and their resulting toxin disorders have been conducted in Iran (19-21). In contrast, only a handful of studies have been carried out on contamination with ZEA and the resulting disorders in humans and animals.

An evaluation of the contamination of human milk samples in Hamadan (Iran) with aflatoxin M1, ochratoxin A, and ZEA showed a mean concentration of 5.98 ng/L of aflatoxin M1, but lower than the detection limit ( < 5 ng/L) for ochratoxin A and ZEA (21). ZEA was also reported in 55% of tested refined maize oil samples in Tehran by high-performance liquid chromatography with a fluorescence detector (22). Another study on detection and quantification of several mycotoxins in Iranian domestic rice reported the presence of 20 fungal metabolites and toxins, among them ZEA isolated from 29.2% of the rice samples (23).

According to this letter, there is not sufficient knowledge about the ZEA fungal toxin and its role in complications related to the human female reproductive system and possible male infertility, especially in infertility specialists. Further experimental dietary and clinical studies on laboratory animals are recommended to investigate this issue.

##  Conflict of Interest

The author declares that there is no conflict of interest.
